# Proof‐of‐concept study of artificial intelligence‐assisted review of CBCT image guidance

**DOI:** 10.1002/acm2.14016

**Published:** 2023-05-10

**Authors:** Jack Neylon, Dishane C. Luximon, Timothy Ritter, James M. Lamb

**Affiliations:** ^1^ Department of Radiation Oncology, David Geffen School of Medicine University of California Los Angeles California USA; ^2^ Department of Medical Physics Virginia Commonwealth University Richmond Virginia USA

**Keywords:** artificial Intelligence, CBCT, IGRT, quality control

## Abstract

**Purpose:**

Automation and computer assistance can support quality assurance tasks in radiotherapy. Retrospective image review requires significant human resources, and automation of image review remains a noteworthy missing element in previous work. Here, we present initial findings from a proof‐of‐concept clinical implementation of an AI‐assisted review of CBCT registrations used for patient setup.

**Methods:**

An automated pipeline was developed and executed nightly, utilizing python scripts to interact with the clinical database through DICOM networking protocol and automate data retrieval and analysis. A previously developed artificial intelligence (AI) algorithm scored CBCT setup registrations based on misalignment likelihood, using a scale from 0 (most unlikely) through 1 (most likely). Over a 45‐day period, 1357 pre‐treatment CBCT registrations from 197 patients were retrieved and analyzed by the pipeline. Daily summary reports of the previous day's registrations were produced. Initial action levels targeted 10% of cases to highlight for in‐depth physics review. A validation subset of 100 cases was scored by three independent observers to characterize AI‐model performance.

**Results:**

Following an ROC analysis, a global threshold for model predictions of 0.87 was determined, with a sensitivity of 100% and specificity of 82%. Inspecting the observer scores for the stratified validation dataset showed a statistically significant correlation between observer scores and model predictions.

**Conclusion:**

In this work, we describe the implementation of an automated AI‐analysis pipeline for daily quantitative analysis of CBCT‐guided patient setup registrations. The AI‐model was validated against independent expert observers, and appropriate action levels were determined to minimize false positives without sacrificing sensitivity. Case studies demonstrate the potential benefits of such a pipeline to bolster quality and safety programs in radiotherapy. To the authors’ knowledge, there are no previous works performing AI‐assisted assessment of pre‐treatment CBCT‐based patient alignment.

## 1 INTRODUCTION

As technology and techniques for radiotherapy treatment have evolved, the prevalence of image guidance has increased. Since image‐guidance is the final step in the radiotherapy workflow prior to initiating beam delivery, any error during pre‐treatment imaging and positioning can severely impact treatment.^1^, ^2^ As such, multiple levels of quality assurance have been implemented which require human observers to review pre‐treatment image registrations. Therapists review all registrations at the console during treatment, physicians review and approve all image guidance daily, and physicists typically perform some level of review during weekly chart checks. With the increased use of image‐guidance, the workload for each of these reviewers increases in kind, carrying a danger of inducing fatigue in the reviewer and allowing errors to pass by undetected.^3^ Quantifying registration performance has historically been a difficult task.^4^, ^5^ Intensity‐based metrics (such as correlation coefficient) and feature‐based metrics (such as mutual information) have many limitations, including sensitivity to intensity range differences and artifacts. In addition, most onboard tools to assist in pre‐treatment image registration rely on these metrics, so they offer little additional benefit as a secondary safety layer.

Recent years have seen a significant effort to apply artificial intelligence (AI) and deep learning techniques in radiotherapy with the aim to improve overall quality and efficiency by utilizing the abundance of prior data to standardize and optimize steps in the radiotherapy workflow. Recent publications detail the efforts in the areas of automatic segmentation, treatment planning, treatment optimization, patient‐specific QA, treatment log analysis, and plan adaptation.^6^, ^7^

There remains a significant gap in the radiotherapy workflow where AI and deep learning have not yet been applied—image review. As discussed in the previous paragraph, most current applications are built on the treatment planning data to include dose distributions, structure sets, and dose volume constraints. McNutt et al. expanded the scope and discussed the application of big data for QA purposes, particularly for identifying anomalies.^6^ However, patients continue producing data throughout the entire course of treatment, and more so now than ever with the increased reliance on image‐guided radiotherapy (IGRT) techniques.

In 2020, the American Association of Physicists in Medicine published the findings of their Task Group 275, on effective strategies for physics plan and chart review.^8^ In this report, they recommend that software vendors develop methods to automate chart reviews, and “highlight items that are difficult to check and review.” Additionally, AAPM's recently published practice guidelines for plan and chart review emphasizes the safe application of computer‐aided programs by ‘calling special attention to missed or mismatched items’ but should not fully replace a thorough and robust chart review.^9^

With most of the field transitioned to electronic medical records (EMR), tools have been developed to automate routine chart checks—comparing logistical data within the database to identify and highlight discrepancies.^10^, ^11^, ^12^ To the authors’ knowledge, there is no current tool available on the market to provide an automated, independent evaluation of the pre‐treatment image‐guided patient alignment and anatomy‐of‐the‐day.

In this work, we examine the implementation of a deep learning algorithm as a decision‐support tool for image review in weekly physics chart checks. Algorithms were previously developed for the detection of IGRT setup errors such as misalignments of 1−2 cm or more, alignments to the incorrect vertebral body, and anatomic misidentifications using IGRT images.^13^, ^14^, ^15^ These algorithms were originally developed to detect rare but serious gross errors and return a misalignment score based on similarity of the aligned IGRT image with the planning CT, accounting for applied IGRT shifts. An appropriately high threshold value of the misalignment score detects gross errors with high sensitivity and specificity. Recently, we hypothesized that an intermediate threshold could be used to differentiate perfectly aligned cases needing minimal human review, from imperfectly aligned cases that, while not gross errors, require human review, clinical judgment, and potentially remediating actions. In this manuscript, we evaluate a working prototype of such a system. We discuss considerations of clinical implementation, including strategy, validation, impact, effectiveness, and efficiency.

The following points summarize the main contributions of this study:

1. We present the methodology of implementation of an emerging AI‐based error detection tool for automatic assessment of pre‐treatment imaging alignment and validated its clinical utility against expert observers, which is a significant and necessary step toward clinical adoption.
2. While the development of the AI‐based tool was presented in a prior publication, its evaluation was only performed using simulated off‐by‐one vertebral body misalignments in the thoracic and lumbar regions. This study is an expansion of the prior work by including models which deal with setup errors in additional treatment regions. Furthermore, by including case reviews using select data presentation modes, we showcased the supplemental benefits the tool can offer during quality assurance image reviews in addition to gross setup error detection.

## 2 METHODS

### 2.1 Error detection algorithm

Our group has developed a convolutional neural net‐based algorithm designed to verify agreement between online CBCT images and the registered planning CTs.^15^ The algorithm was trained to detect positioning misalignments such as translational errors of 1 cm or more and off‐by‐one vertebral body errors. Initial testing demonstrated an ability to distinguish perfectly aligned patients from alignments that were sub‐optimal due to a variety of challenges including patient weight loss or gain, bladder. and bowel filling changes versus simulation, and body pose changes such as spine curvature or hip tilt.

Algorithm design and development is briefly described in the following section. Further details are available elsewhere.^15^ Separate neural net models were used for each body site: head and neck, thoracic‐abdominal (TA), and pelvic. Each anatomy‐specific model follows a 4‐level Dense‐Net architecture.^16^ Each model takes as input 3 orthogonal planes of the CBCT and corresponding planes from the planning CT according to the rigid registration used to align the patient at time of treatment. For the head & neck (HN) and TA models, the orthogonal 2D slices were automatically extracted about a point within the vertebral column which was obtained from either the spinal canal structure set within the RTSTRUCT file or a dedicated spinal canal segmentation model. For pelvis (PL) cases, as the registration is highly reliant on the tumor position and fiducial markers around it, the orthogonal planes were consequently extracted about the centroid of the primary target volume (PTV) or treatment isocenter.

The output of the model is a misalignment likelihood prediction with 0 indicating perfect alignment and 1 indicating the highest probability of misalignment. The models were trained and validated on a total of 6376 image pairs obtained from 680 patients as part of an IRB‐approved retrospective study at UCLA and Virginia Commonwealth University. Further details are provided in Table SA1. Training images consisted of clinically aligned cases (representing “no error”) and simulated misalignments obtained by translating images away from the correctly aligned state. For the head and neck and PL models, 10 mm translations in multiple directions were used. For the TA model, off‐by‐one vertebral body misalignments (superior and inferior) were used.

An anatomical region labeling (ARL) model^17^ was used to stream CBCT images to the appropriate anatomy‐specific pipeline. The ARL model was trained and tested on the UCLA patient datasets. For each scan, the coronal slice taken from the middle of the CBCT scan was extracted and inputted to the ARL model. This particular plane was selected as it contains overall body shape information and organ structures which could be useful for the anatomy region classification. The ARL model was based on the Dense‐Net architecture which made use of densely contracting paths to capture contextual information from a selected coronal slice from the CBCT scan before outputting a probability for each of the three anatomical regions. During model training, the model was validated using the CBCT scans in the validation datasets, and the validation accuracy was used to test for model convergence.

### 2.2 Proof‐of‐concept implementation

In accordance with the recommendation in the AAPM practice guidelines^7^ the intent of integrating automated software‐based supervision into the radiotherapy QA process was not to supplant weekly physics chart reviews, but to supplement it and aid in identifying cases which may need closer inspection. As such, our implementation strategy was to highlight a subset of cases each day for in‐depth investigation.

**FIGURE 1:**
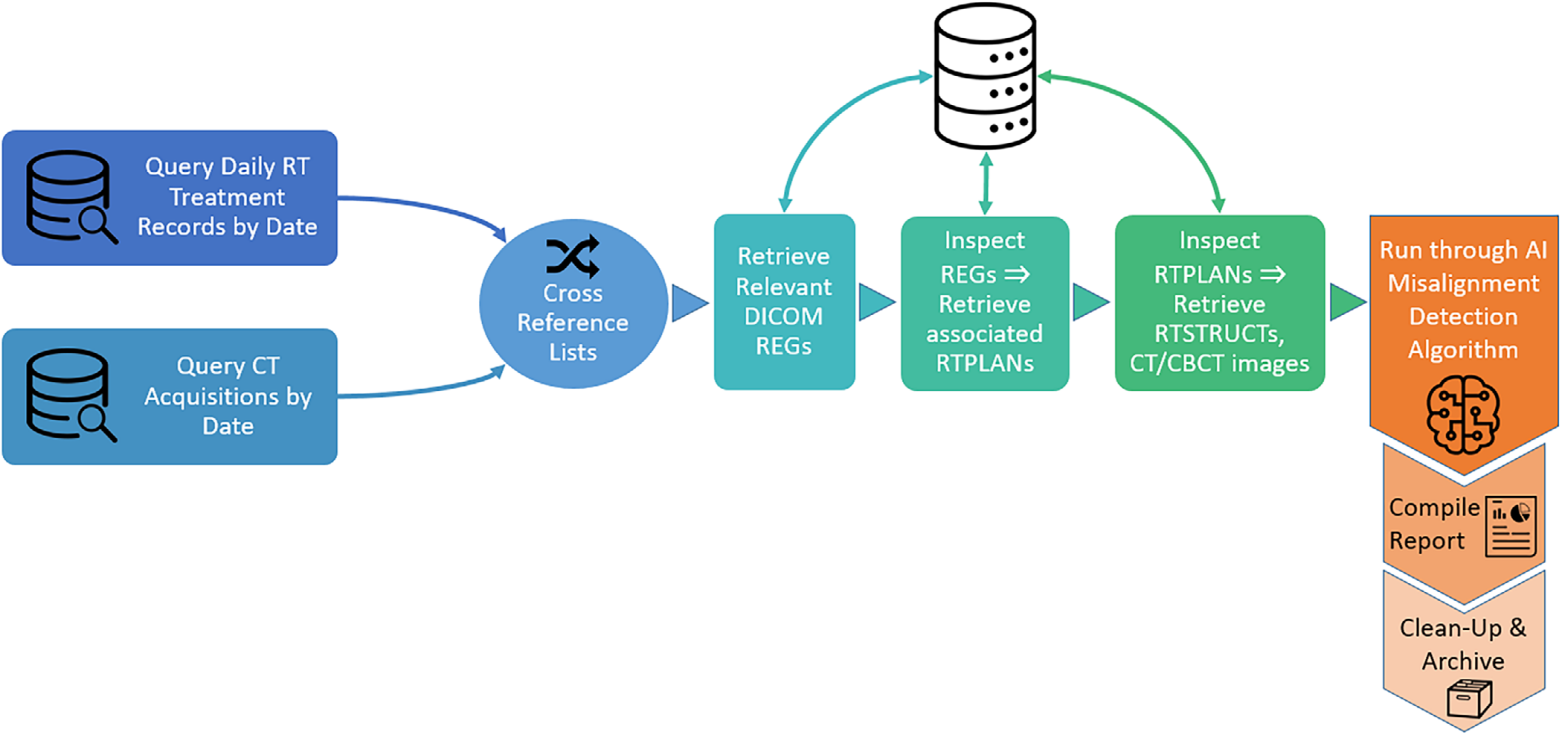
Workflow illustration of the automated pipeline.

The proof‐of‐concept implementation was developed with Python scripting and utilized a DICOM networking protocol to query and retrieve data from the clinical record and verify (R&V) system. The prototype system was developed to interface with the ARIA R&V system (Varian Medical Systems, Palo Alto, CA) using the pynetdicom^1^ Python package. Results were compiled into daily reports and aggregated into an interactive dashboard. The workflow is illustrated in Figure 1, and the nine modular components can be described as follows: (1) Query the clinical database for a list of daily treatments. (2) Query the clinical database for a list of daily cone‐beam CT (CBCT) acquisitions. (3) Cross‐reference lists to identify patients for analysis. (4) Retrieve relevant DICOM Registrations (REGs) for identified patients. (5) Inspect REGs, and retrieve referenced RTPlans. (6) Inspect RTPlans, and retrieve associated RTStructs, planning CT images, and CBCT images. (7) Run an AI‐based misalignment model on each dataset. (8) Compile predictions into a daily report. (9) Archive intermediate results, logs, and remove temporary files.

A web‐based dashboard was implemented to facilitate access to the daily reports, where results are sorted by prediction value and highlight the most likely misalignments from the previous days’ treatments. Additional features were developed to facilitate a superficial review, but with the stipulation that full in‐depth review should still be performed using the R&V system's built‐in tools. These features included single‐slice CT‐CBCT fusions along each axis to quickly identify if there was an issue with the data retrieval or preprocessing. The user may also scroll quickly through the patient's past treatments to compare the registrations or look for trends. Lastly, summary statistics are compiled and charted, allowing breakdown by machine, date, treatment site, prediction value, and so forth. to help explore overall data trends in the clinic.

The AI‐algorithm scored the likelihood of a misalignment between 0 and 1: 0 being most unlikely and 1 being most likely for a misalignment. An initial threshold was implemented at 0.1 with the intention of flagging 5%−10% of cases for further investigation.

A clinical validation study was performed to assess the suitability of the chosen threshold and the ability of the algorithm to identify setup images that might, in the clinical judgment of a medical physicist, require further investigation. In an IRB approved study, the proof‐of‐concept implementation was run retrospectively on data from treatments between 7 February 2022 and 10 May 2022. Treatments were performed on four machines: 1 NovalisTx, 1 NovalisSTx, and 2 TrueBeams (Varian Medical Systems, Palo Alto, CA). A total of 1357 treatment cases comprising registered (post‐shift) CBCT images, were analyzed from 197 unique patients.

From this data, 100 cases were selected for expert review based on the following criteria: 50 cases having the highest misalignment scores (lowest model prediction – 0.495), and 50 randomly selected cases from the rest of the population. Each case was from a unique patient to avoid image‐score correlations. The list of cases was then randomly permutated. Three independent observers, board certified medical physicists, manually reviewed each of the 100 cases and scored them from 1 to 4. Review criteria were based on clinical action levels, and were scored based on overall alignment, taking into account the reviewer's estimation of whether a given target should be aligned to soft tissue or bone. The numerical review score was defined as follows: 1 showing a perfect match of target and surrounding anatomy; 2 showing some deviation, but clinically acceptable; 3 showing enough deviation to require further investigation, and 4 showing significant deviation that would preclude treatment until investigation is resolved.

The mean values of the reviewer's score were correlated with the AI prediction. Algorithm performance at discriminating cases with mean overall score greater than 2 (i.e., action level requiring investigation) was quantified using a receiver operating characteristic (ROC) curve. As a random selection process was applied to the stratified patient dataset (50 highest‐scoring cases, then 50 randomly chosen from the remaining 147 cases), the weight of each sample was calculated by taking the inverse of the sample proportion from each of the two strata. The weight of a sample found in the lower 147 cases was 147/50 = 2.97. The samples found in the 50 highest‐scoring cases were assigned a weight of 1 as the whole population was used in the analysis. Using these weights, the weighted sensitivity and specificity were calculated and used to build a weighted ROC curve. This weighting method has been established as an effective method to extrapolate the findings from a sample study to the entire dataset.^18^ Additionally, selected representative cases were examined further with temporal trend‐line plots.

## 3 RESULTS

### 3.1 Error detection algorithm

The region receiving the highest output score from the anatomic region labeling (ARL) model was chosen as the final model prediction and was compared to the ground truth region. The accuracy of the ARL model was found to be 99.4% on the test dataset, with two misclassifications out of 1611 coronal slices from 256 patients. For the TA, HN, and PL error‐detection models, the area under curve (AUC) of the ROC curve was used to assess the performance of each model in classifying the registrations from their respective test dataset.^19^ Due to the rarity of registration errors in the clinic, a minimum threshold value yielding a specificity of at least 99% was selected. Using this threshold, the sensitivity was calculated for each model. The performance of each model is presented in Table 1.

**TABLE 1:** Performance of each model in our error detection pipeline

**Model**	**Error type**	**AUC**	**Specificity**	**Sensitivity**
Thoracic‐abdominal (TA)	Off‐by‐one vertebral body misalignment	99.4%	99%	95%
Head & neck (HN)	10 mm shift	99.6%	99%	89%
Pelvis (PL)	10 mm shift	99.2%	99%	89%

Abbreviation: AUC, area under curve.

### 3.2 Proof‐of‐concept implementation

Data was automatically collected and processed over a 45‐day period, resulting in 1357 registrations from 197 unique patients being analyzed. The distribution of registrations by anatomical region included 506 HN, 464 PL, and 387 TA. To ensure the validity of our proof‐of‐concept study, the patient population for this study was kept independent of the patient population used for model training and validation, described in Table SA1.

**FIGURE 2:**
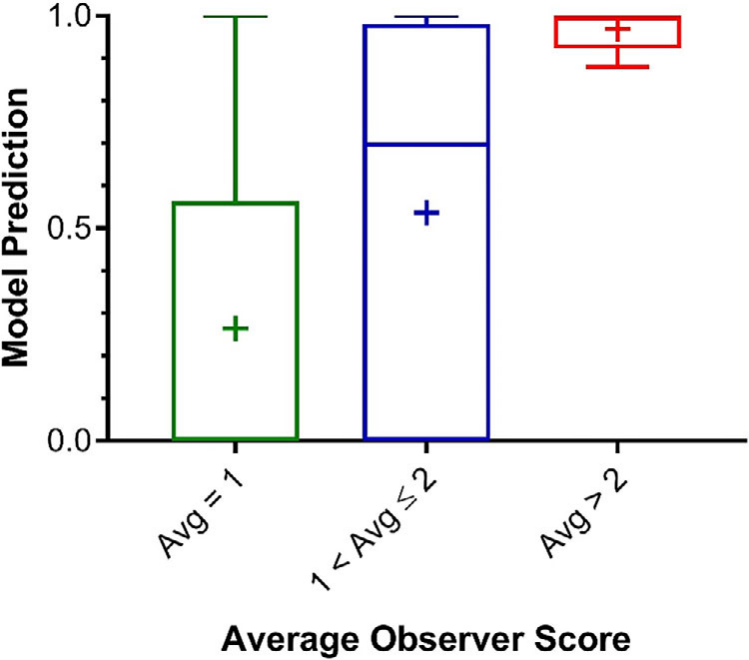
Box and whisker plot to show the distribution of the model predictions, grouped by average observer score. The box shows the median, 25th percentile, and 75th percentile, while the whisker shows the minimum and maximum values. The cross within each box represents the mean model prediction for the respective group.

The full distribution of observer scores obtained from the 100 case reviews are shown in Table SB1. Figure 2 illustrates the relationship between the observer scores and model predictions for the 100 registrations in the validation set. Cases were binned by average observer score, and a box and whisker plot were constructed to show the distribution of the model predictions for each group.

After confirming the correlation of model predictions to observer scores, the focus shifted to determining an optimal threshold prediction when flagging cases for further investigation. Ideally, a threshold could be found that would catch all cases tagged by the observers as less than ideal, while also minimizing false positives. It was considered a priority to minimize false positives to limit the time required for daily review and avoid inducing alarm fatigue.

**FIGURE 3:**
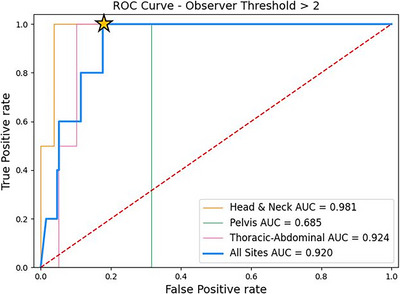
Weighted receiver operating characteristic (ROC) curves obtained using a mean observer threshold >2. The bold blue curve represents the results from the entire 100 patient validation dataset, and the other curves represent the results from each respective anatomical region only. The area under curve (AUC) is also given for each curve. The model prediction threshold (0.87) leading to a sensitivity of 100% and a specificity of 82% (depicted by the yellow star) was obtained and used for further analysis.

Figure 3 plots the ROC curves for the validation set, as well as individual ROC curves for each anatomical region. True positives were categorized as cases where the mean observer score was greater than 2, where a score of 2 was considered clinically acceptable but not perfect. From the ROC analysis, it is apparent that using a threshold of 0.87 achieves 100% sensitivity while minimizing false positives. Applying this threshold to the validation data set of 100 cases, 40 would be flagged for further investigation. Note, this proportion is not reflective of a broader patient population since the validation dataset included the 50 cases receiving the highest model prediction scores (mean prediction of 0.91 [top 50] vs. 0.05 [the rest]—*p*‐value < 0.0001). Inspecting the observer scores for the stratified validation dataset shows an average of 1.65 ± 0.51 for model predictions ≥0.87, and 1.33 ± 0.33 for model predictions <0.87. A two‐tailed t‐test results in a *p*‐value of 0.0002 between these cohorts.

## 4 DISCUSSION

To explore how the proposed tool could potentially impact clinical workflow, we present case studies for discussion. With most clinics transitioned to EMR, it is trivial for software tools to compare values between database entries and highlight discrepancies. The task is more difficult and nuanced for image review, and requires both broad clinical knowledge and patient‐specific insight to determine relevance and priority. The proposed deep learning pipeline aims to provide a quantitative analysis of daily pre‐treatment CBCT alignment, and has the potential to facilitate the recognition of anomalies. Thresholding may be used to identify a manageable number of datasets for manual review. A trendline may provide added value when used in parallel to the hard‐thresholding method.

**FIGURE 4:**
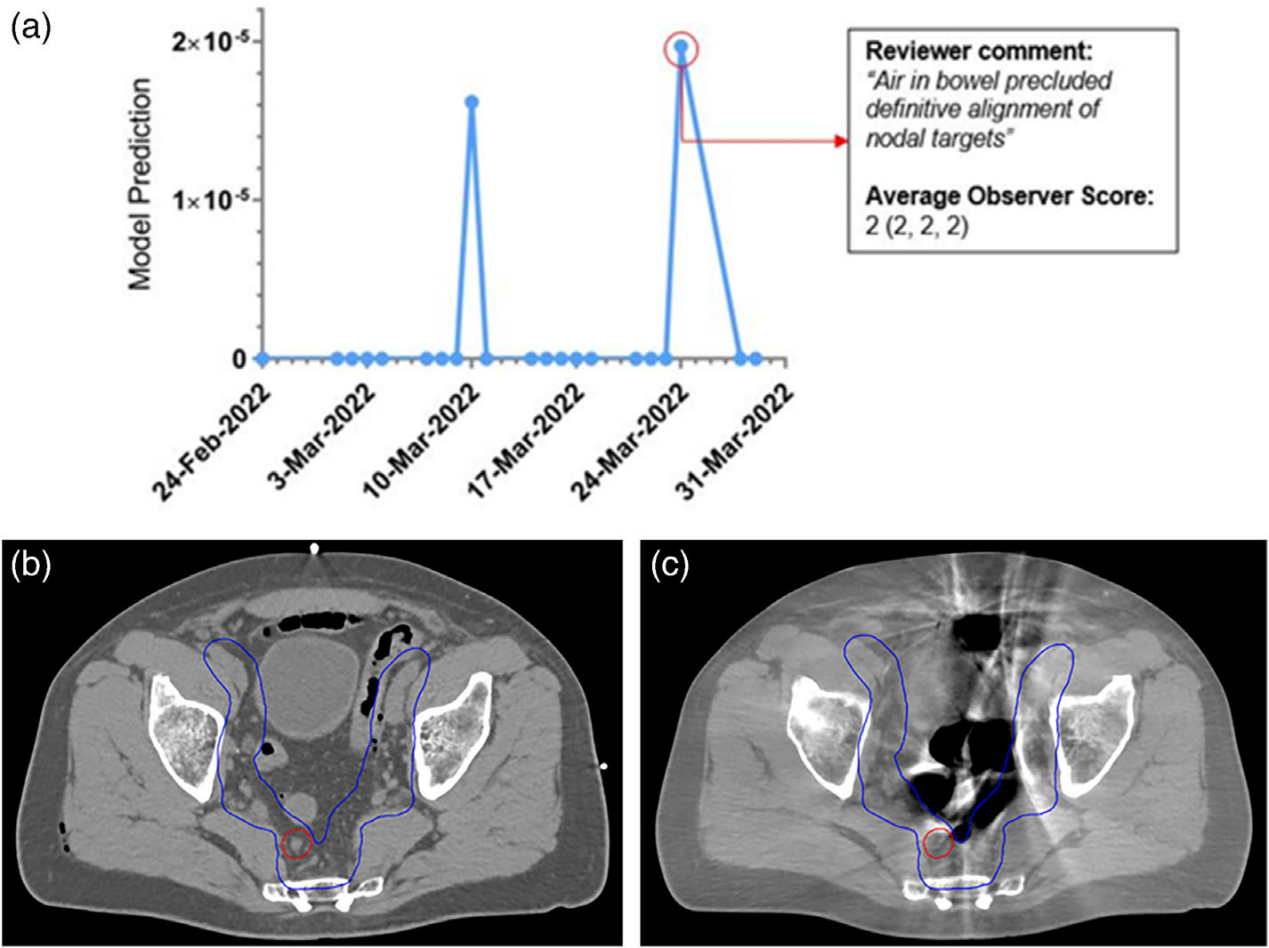
This diagram depicts a case where the deep learning model would not have flagged the registration due to the low prediction score (2 × 10^−5^). However, the trendline in (a) suggests a relatively high escalation in the model prediction on that particular day (24 March 2022) as compared to the previous treatment days, demonstrating the potential value the trendline can provide when used in parallel to the hard‐thresholding method. Images (b) and (c) are select axial slices from the planning CT and pre‐treatment CBCT (24 March 2022), respectively. The presence of gas in the bowel resulted in artifacts on the CBCT image and inhibits identification of targets within the 50 Gy planning tumor volume (PTV) coverage (blue contour) and the 62.5 Gy gross node PTV coverage (red contour).

One example is illustrated in Figure 4. The timeline in Figure 4a plots the model prediction from each fraction by date over the patient's treatment course. While none of the model prediction scores approach our global threshold to be flagged for further inspection, there are clearly two fractions where the deep learning model identified an increased probability of misalignment. One of these fractions was included in our validation dataset, and an observer commented, “Air in bowel precluded definitive alignment of nodal targets.” Figure 4b,c display the anatomy from this fraction, where increased air cavities in the bowel resulted in artifacts on the CBCT image which made target identification and pre‐treatment setup more difficult.

**FIGURE 5:**
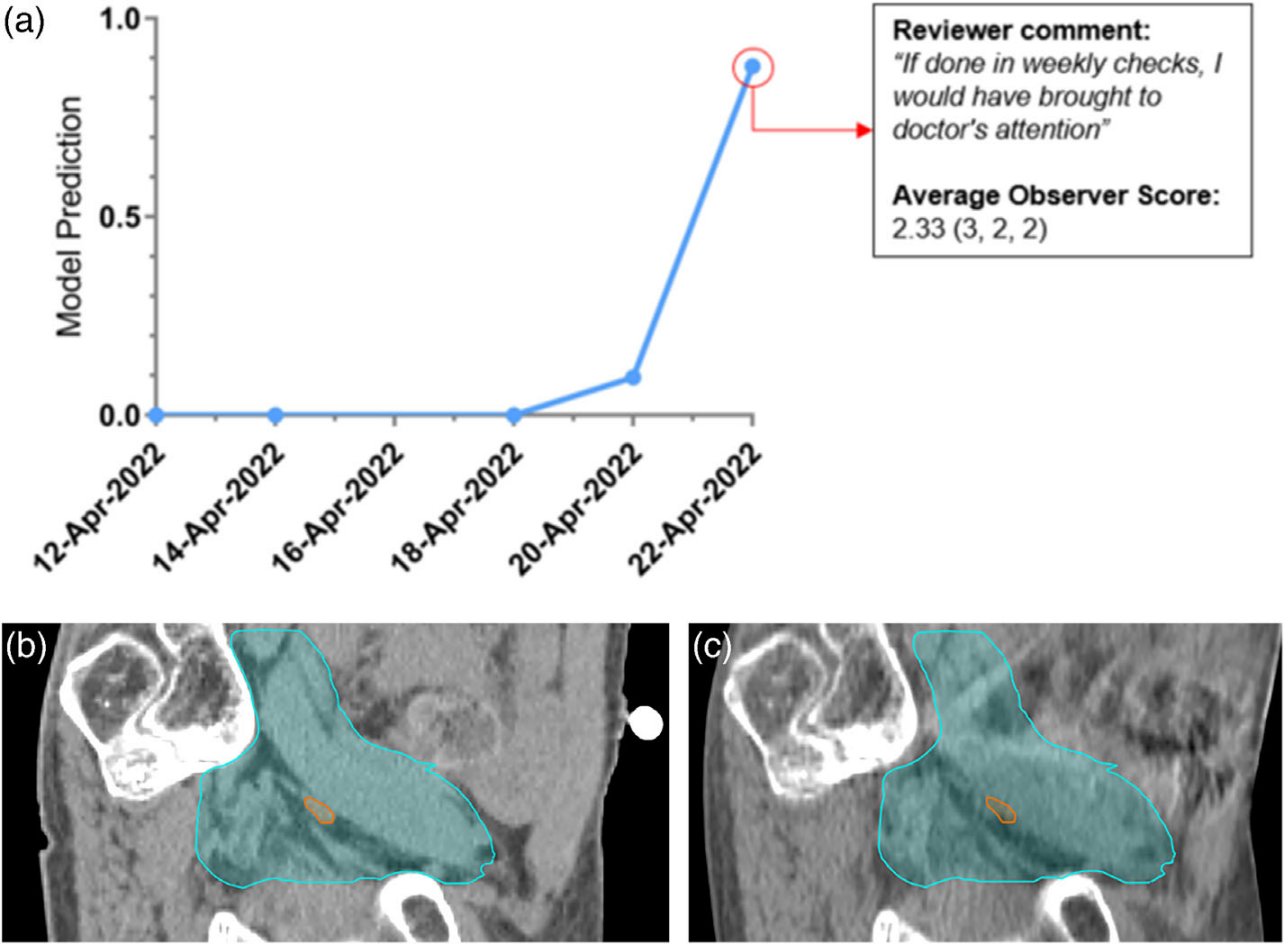
Highlight of a case which obtained a relatively high mean observer score (2.33) and a high model prediction score (0.88). The trendline in (a) shows the model prediction scores of the registrations performed over the course of the patient's treatment. The red circled point represents the case which was reviewed by expert observers for validation. Images (b) and (c) are select sagittal slices from the planning CT and setup CBCT (22 April 2022), respectively. Differences in the 25 Gy planning tumor volume (PTV) coverage (blue contour) and in the gross node PTV coverage (orange contour) can be observed between (b) and (c).

**FIGURE 6:**
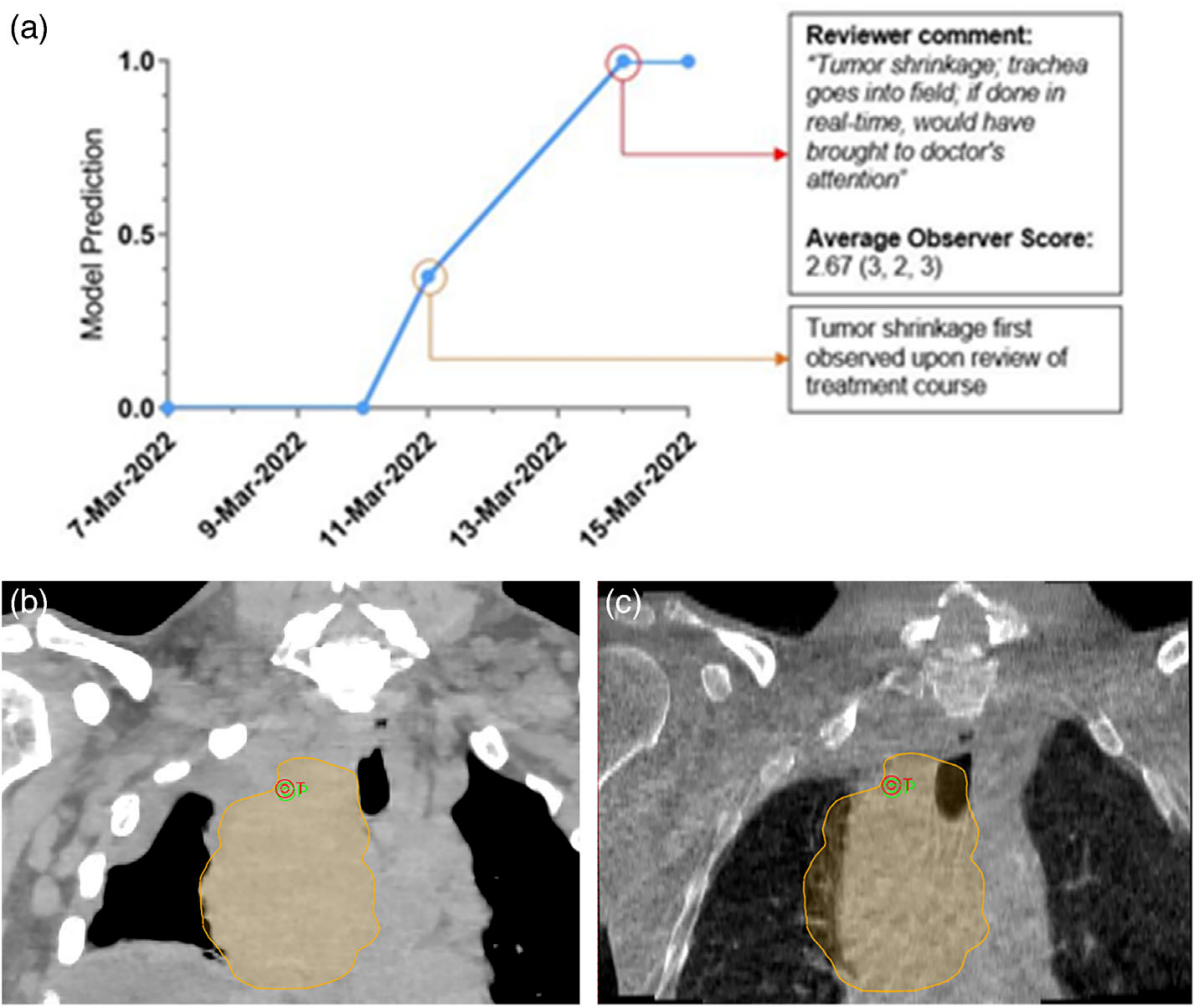
Highlight of a case which obtained a relatively high mean observer score (2.67) and a high model prediction score (1.0). The trendline in (a) shows the model prediction scores of the registrations performed over the course of the patient's treatment. The red circled point represents the case which was reviewed by expert observers for validation. The other registrations were reviewed post‐analysis for comparison. (b) Shows a selected coronal slice from the planning CT, with the planning tumor volume (PTV) shown as the yellow overlay and the treatment isocenter shown as the red target. (c) Shows the pre‐treatment CBCT from 14 March 2022.

Over the course of treatment, gradual anatomical and physiological changes can also make pre‐treatment setup more difficult and may indicate the necessity for intervention. Figures 5 and 6 highlight two such instances.

For the case illustrated in Figure 5, the trendline shows consistently good alignment for the first few fractions, then a relative increase for fraction 4, before a dramatic jump on fraction 5. Reviewing the images, there is a clear shift of the bony anatomy between the plan (Figure 5b) and the CBCT (Figure 5c). Additionally, the lymph node boost volume is difficult to visualize but may be posterior to the nodal PTV in the CBCT image. Fraction 5 was included in the validation dataset, and one of the observers noted, “If done in weekly checks, I would have brought to doctor's attention.”

To contrast the case in Figure 5, which displayed subtle differences that could be attributed to user judgment during registration at the treatment console, the case illustrated in Figure 6 shows drastic anatomic changes. The patient had a large mediastinal mass that shrunk significantly over a 5‐fraction treatment course, as well as pleural effusion that showed improvement. The fourth fraction was included in the validation dataset, and the tumor shrinkage was noted with two observers scoring the case “3” and one commenting, “Tumor shrinkage; trachea goes into field. If done in real‐time, would have brought to doctor's attention.” The model prediction timeline shows a clear progression, for longer treatment courses or more drastic anatomic changes, this could be a valuable tool to anticipate re‐planning.

**FIGURE 7:**
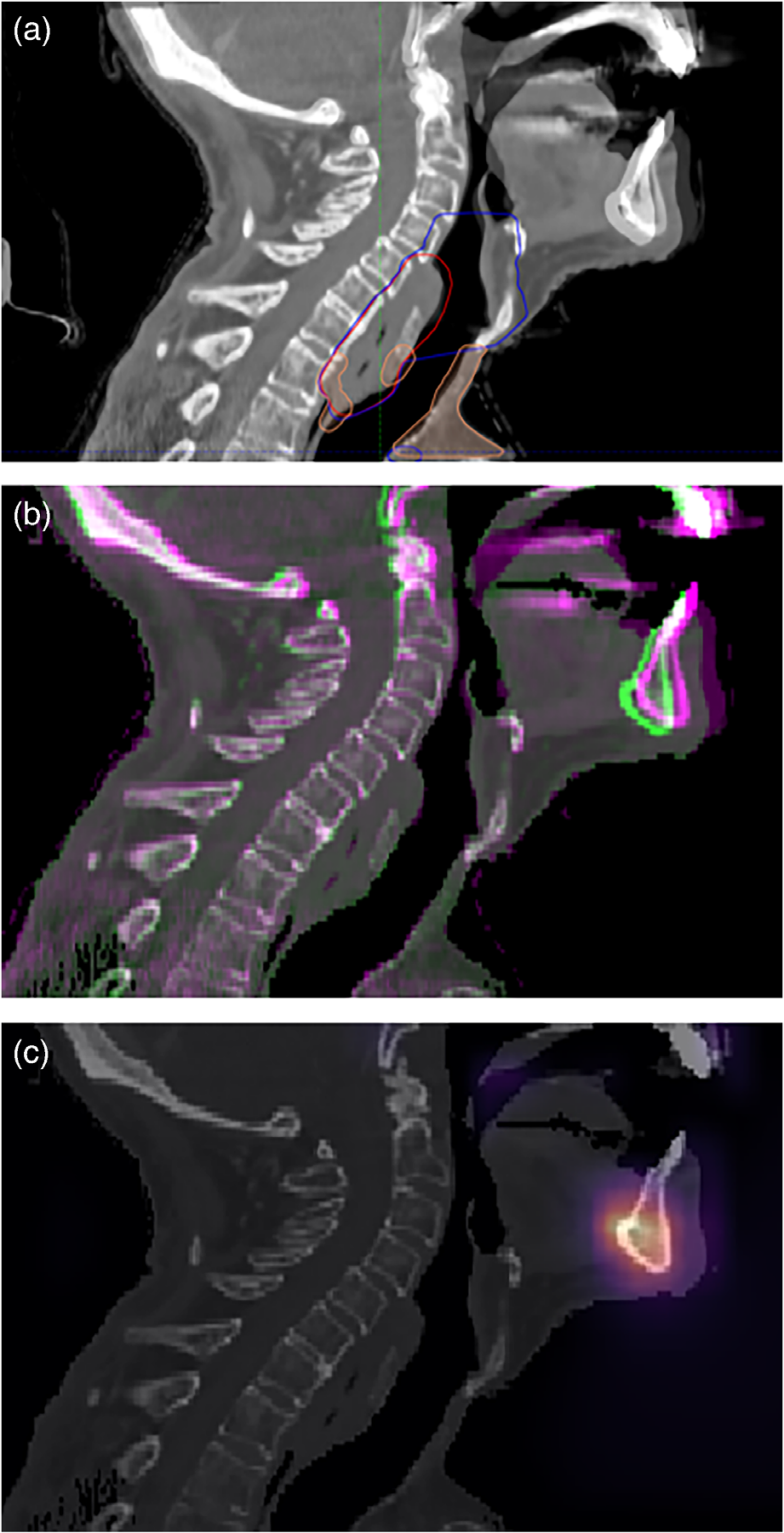
Debugging the misalignment predictions with a model activation heatmap. (a) Shows the CT‐CBCT fusion with target contours overlaid, (b) shows a colormap fusion of planning CT (green) and CBCT (purple), and (c) shows the activation map of our deep learning model overlaid on the planning CT. The mandible is clearly misaligned in this case, and the heatmap shows a hotspot for model activation at the mandible. This feature provides an avenue for the user to better understand the reason behind the model's misalignment predictions.

While a deep learning model can identify and quantify differences between the planning CT image and the daily CBCT image, a human observer is still required to review the flagged cases and judge whether the differences are clinical relevant and actionable. To further aid the observer, a debugging tool was incorporated into the proof‐of‐concept pipeline, which overlays a heatmap of the model activation on the patient anatomy, indicating the areas contributing to the misalignment prediction. Figure 7 demonstrates this debugging tool on a bilateral neck case. Figure 7a shows a sagittal slice the CT‐CBCT fusion with target contours overlaid. A colormap fusion is displayed in Figure 7b, with the planning CT in the green channel and the CBCT in the red and blue channels. The activation heatmap is overlaid on the planning CT in Figure 7c. This heatmap could be used as a debugging tool for the end‐user to visualize the model prediction.

The case studies above illustrate the potential uses of the pipeline. Nevertheless, some limitations apply to the current proof‐of‐concept system. The deep learning model, at least in its current iteration, does not incorporate clinical context and determine relevance of a misalignment. Additionally, as with many computer vision tools, it will preferentially focus on high‐contrast image features such as bone and tissue‐air interfaces. Lastly, while intended to be vendor agnostic, utilizing only DICOM networking protocol to interface with the clinical database, the proof‐of‐concept pipeline was developed referencing only a single vendor's DICOM conformance statement and the built‐in DICOM queries reflect that. Connecting to another vendor R&V system, or even a different version of the same vendor's R&V system would almost certainly require further development of the DICOM handling.

## 5 CONCLUSION

Due to time and manpower constraints, it is often not feasible for physicists to perform in depth examination of every pre‐treatment alignment registration. As discussed in the introduction, image guidance is one of the most pivotal steps in the treatment workflow, with a significant risk that a mistake could lead to a mistreatment.

In this work, we presented a proof‐of‐concept clinical implementation of an automated pipeline for AI‐assisted CBCT alignment retrospective review. The purpose of the pipeline was to provide quantitative assessment and visualization tools to improve the efficiency and efficacy of periodic chart reviews. The predictions of the deep learning model were validated against expert observers, and demonstrated that a prediction threshold could be identified to stratify pre‐treatment images with a statistically significant correlation to the observer scores. In addition to the validation study, we demonstrated through anecdotal examples how these tools could be beneficial to the clinical workflow through quantification of daily alignments and patient/plan specific timelines to identify trends and flag anomalies. Effective visualization of this data, made quickly accessible and easily digestible, can expedite the image review component of periodic physics chart checks.

Future work will aim to leverage the speed of deep learning inference to move this system from retrospective to real‐time, integrating directly with the treatment machine to interlock the beam if the AI‐model flags a potential setup misalignment. Such a system would ideally require the operator to either revise the alignment or acknowledge the interlock before proceeding with treatment. An automated AI‐assisted tool could allow for independent, quantitative review of every alignment registration in the future.

## AUTHOR CONTRIBUTIONS

All listed authors provided substantial contributions to the conception and design of this work, the acquisition and interpretation of the data, and the writing and editing of this manuscript.

## CONFLICT OF INTEREST

The authors declare no conflicts of interest.

## Supporting information

Table A1: Description of the dataset used to train the deep‐learning models in the error detection algorithm.
Table A2: Categorization by observer score of the 100 cases stratified by using a model prediction threshold of 0.87
Table B1: Absolute count (N) of the observer scores for each individual expert in our study

## Data Availability

Research data are not available at this time.

## Footnotes

1 https://pydicom.github.io/pynetdicom
