# Supplementary material for: Proof‐of‐concept study of artificial intelligence‐assisted review of CBCT image guidance
Source: J Appl Clin Med Phys. 2023 May 10;24(9):e14016. doi: 10.1002/acm2.14016 (PMC10476980; doi:10.1002/acm2.14016)
Supplement: Supplementary file 1 — Table A1: Description of the dataset used to train the deep‐learning models in the error detection algorithm. Table A2: Categorization by observer score of the 100 cases stratified by using a model prediction threshold of 0.87 Table B1: Absolute count (N) of the observer scores for each individual expert in our study [file ACM2-24-e14016-s001.docx]

| **Table A1:** Description of the dataset used to train the deep-learning models in the error detection algorithm. The dataset was partitioned into a training, validation, and testing set for each treatment site. | | | | | |
| --- | --- | --- | --- | --- | --- |
|  | | **Number of patients** | **CBCT Image Pairs** | | |
|  |  |  | **Total** | **Aligned** | **Misaligned** |
| **Thoracic/Abdominal** | Training (UCLA/VCU) | 374  (304/70) | 1887 (1677/210) | 1139 (1069/70) | 748 (608/140) |
|  | Validation (UCLA/VCU) | 39 (29/10) | 186 (156/30) | 108 (98/10) | 78 (58/20) |
|  | Testing (UCLA/VCU) | 67 (47/20) | 303 (243/60) | 169 (149/20) | 134 (94/40) |
| **Head & Neck** | Training  (UCLA only) | 60 | 912 | 456 | 456 |
|  | Validation (UCLA only) | 10 | 76 | 38 | 38 |
|  | Testing (UCLA only) | 30 | 354 | 177 | 177 |
| **Pelvis** | Training (UCLA only) | 60 | 1600 | 800 | 800 |
|  | Validation (UCLA only) | 10 | 262 | 131 | 131 |
|  | Testing (UCLA only) | 30 | 796 | 398 | 398 |
| *CBCT: Cone-Beam Computed Tomography;* | | | | | |

| **Table A2:** Categorization by observer score of the 100 cases stratified by using a model prediction threshold of 0.87 | | |
| --- | --- | --- |
|  | **Prediction ≥ 0.87** | **Prediction < 0.87** |
| **Number of cases with mean observer score ≤ 2** | **35** Total | **60** Total |
| **Number of cases with mean observer score > 2** | **5** Total | **0** |
| *HN: Head & Neck, PL: Pelvis, TH: Thoracic-abdominal* | | |

| **Table B1:** Absolute count (N) of the observer scores for each individual expert in our study. | | | |
| --- | --- | --- | --- |
| **Observer Score (X)** | **N (Observer 1)** | **N (Observer 2)** | **N (Observer 3)** |
| 1 ≤ X < 2 | 65 | 77 | 35 |
| 2 ≤ X < 3 | 32 | 20 | 59 |
| 3 ≤ X < 4 | 3 | 2 | 6 |
| X = 4 | 0 | 1 | 0 |
